# Advanced Oxidation Protein Products-Modified Albumin Induces Differentiation of RAW264.7 Macrophages into Dendritic-Like Cells Which Is Modulated by Cell Surface Thiols

**DOI:** 10.3390/toxins9010027

**Published:** 2017-01-10

**Authors:** Silvano Garibaldi, Chiara Barisione, Barbara Marengo, Pietro Ameri, Claudio Brunelli, Manrico Balbi, Giorgio Ghigliotti

**Affiliations:** 1 Division of Cardiology, IRCCS University Hospital San Martino, Research Centre of Cardiovascular Biology, University of Genova, Genova 16132, Italy; 700086@unige.it (S.G.); 700915@unige.it (C.B.); pietroameri@unige.it (P.A.); bc@unige.it (C.B.); manrico.balbi@unige.it (M.B.); 2 Department of Experimental Medicine, University of Genova, Genova 16132, Italy; Barbara.Marengo@unige.it

**Keywords:** AOPP, oxidative stress, dendritic cells, thiols, NAC

## Abstract

Local accumulation of Advanced Oxidation Protein Products (AOPP) induces pro-inflammatory and pro-fibrotic processes in kidneys and is an independent predictor of renal fibrosis and of rapid decline of eGFR in patients with chronic kidney disease (CKD). In addition to kidney damage, circulating AOPP may be regarded as mediators of systemic oxidative stress and, in this capacity, they might play a role in the progression of atherosclerotic damage of arterial walls. Atherosclerosis is a chronic inflammatory disease that involves activation of innate and adaptive immunity. Dendritic cells (DCs) are key cells in this process, due to their role in antigen presentation, inflammation resolution and T cell activation. AOPP consist in oxidative modifications of proteins (such as albumin and fibrinogen) that mainly occur through myeloperoxidase (MPO)-derived hypochlorite (HOCl). HOCl modified proteins have been found in atherosclerotic lesions. The oxidizing environment and the shifts in cellular redox equilibrium trigger inflammation, activate immune cells and induce immune responses. Thus, surface thiol groups contribute to the regulation of immune functions. The aims of this work are: (1) to evaluate whether AOPP-proteins induce activation and differentiation of mature macrophages into dendritic cells in vitro; and (2) to define the role of cell surface thiol groups and of free radicals in this process. AOPP-proteins were prepared by in vitro incubation of human serum albumin (HSA) with HOCl. Mouse macrophage-like RAW264.7 were treated with various concentrations of AOPP-HSA with or without the antioxidant *N*-acetyl cysteine (NAC). Following 48 h of HSA-AOPP treatment, RAW264.7 morphological changes were evaluated by microscopic observation, while markers of dendritic lineage and activation (CD40, CD86, and MHC class II) and allogeneic T cell proliferation were evaluated by flow cytometry. Cell surface thiols were measured by AlexaFluor-maleimide binding, and ROS production was assessed as DCF fluorescence by flow cytometry. HSA-AOPP induced the differentiation of RAW264.7 cells into a dendritic-like phenotype, as shown by morphological changes, by increased CD40, CD86 and MHC class II surface expression and by induction of T cell proliferation. The cell surface thiols dose dependently decreased following HSA-AOPP treatment, while ROS production increased. NAC pre-treatment enhanced the amount of cell surface thiols and prevented their reduction due to treatment with AOPP. Both ROS production and RAW264.7 differentiation into DC-like cells induced by HSA-AOPP were reduced by NAC. Our results highlight that oxidized plasma proteins modulate specific immune responses of macrophages through a process involving changes in the thiol redox equilibrium. We suggest that this mechanism may play a role in determining the rapid progression of the atherosclerotic process observed in CKD patients.

## 1. Introduction

Advanced Oxidation Protein Products (AOPP) are oxidative stress biomarkers that are initially found at high levels in the plasma of patients with chronic kidney disease (CKD). Furthermore, it was shown that AOPP correlate with creatinine clearance and, since then, they have been considered a marker of renal damage progression [1]. AOPP include oxidative modifications of proteins that mainly occur through myeloperoxidase (MPO)-derived hypochlorite (HOCl) production [2]. 

AOPP concentrations were found to be the highest in hemodialysis patients followed by patients on peritoneal dialysis. Similarly, AOPP levels in non-dialyzed pre-terminal renal failure patients were also increased as compared to healthy controls, which led to AOPP being included in the family of uremic toxins. Moreover, the ability to activate phagocytes and to induce production of cytokines such as interleukin-6 (IL6), tumor necrosis factor alpha (TNF), and interleukin-1beta (IL1b) suggests that AOPP act as pro-inflammatory mediators [3,4]. 

Besides their role in the progression of uremia, AOPP are involved as mediators of systemic oxidative stress and of inflammatory processes in several clinical conditions such as diabetes mellitus and its renal complications, systemic sclerosis and coronary artery disease [5,6,7,8]. 

AOPP have been linked to progression of atherosclerosis and to the occurrence of cardiovascular events [9] as is also observed with other protein-bound uremic toxins [10].

Both the generation of free radicals in cells and the inflammatory response induced by AOPP are important drivers of the progression of atherosclerosis and as triggers of cardiovascular disease (CVD) events. As plasma protein bound moieties, AOPP act at the circulating level as well as on resident cells. The interaction and effect on innate immune cells, such as neutrophils, monocytes, macrophages and dendritic cells (DCs) have come under careful scrutiny. It has been demonstrated that immune cells, such as monocytes and neutrophils are activated by AOPP. AOPP induce oxidative bursts in monocytes and in neutrophils in vitro. Oxygenation activities of NADPH oxidase and Myeloperoxidase in polymorphonuclear cells and monocytes increased in vitro in a dose-dependent manner following treatment with human serum albumin (HSA)-AOPP [11,12]. Recruitment of mononuclear phagocytes occurs during inflammatory processes and gives rise to differentiated cells including inflammatory DCs with different functions such as antigen presentation to effector T cells. Thus, DCs in secondary lymphoid organs work toward the induction and maintenance of systemic immunity. However, due to their plasticity, monocytes, macrophages and DCs surface markers and functions largely overlap, and, moreover, an excessive reaction to systemic antigens may spread responses therefore potentially damaging arterial vessel walls and body tissues [13,14]. 

Besides residing in lymphoid organs, DCs are present in most peripheral tissues in the body and are activated by inflammatory signals. In turn, they activate T cells, thus providing a bridge between innate and adaptive immunity. The main activity of DCs consists in the presentation of antigens to T cells in the periphery, thereby contributing to protection against pathogens. This pathway becomes overstimulated in chronic pro-inflammatory conditions, and, in this case, in addition to linking adaptive and innate immunity, DCs also act in an antigen-independent fashion, thus bypassing protective immunity and promoting tissue fibrosis, as occurs in advanced renal diseases [15,16,17].

Redox status has recently been implicated in cell signaling, and post-translational modifications of proteins may participate in gene expression control and metabolism regulation. An oxidizing environment may act as an activator of both circulating and resident immune cells. The redox equilibrium and a non-oxidizing environment are key factors in maintaining protective (functional) cell immunity: disturbances in this homeostatic mechanism may trigger altered inflammatory and immune responses. Albumin is considered the main antioxidant molecule in plasma due to its abundance in thiol groups [18]. Thiol groups participate in cellular redox homeostasis and contribute to regulating immune functions. There is evidence that AOPP are oxidative stress biomarkers, but that they are also circulating moieties that affect leukocytes. Moreover, hypochlorite-modified proteins have been found in atherosclerotic lesions. The aim of this work is to evaluate whether AOPP-protein might induce the differentiation of mature macrophages into DCs and whether cell surface thiol groups are involved in this process. The efficacy of the *N*-acetyl cysteine (NAC) antioxidant in modulating these processes has also been tested.

## 2. Results

### 2.1. AOPP Induced Morphologic Changes in RAW264.7 Cells toward DC-Like Cells

**Figure 1:**
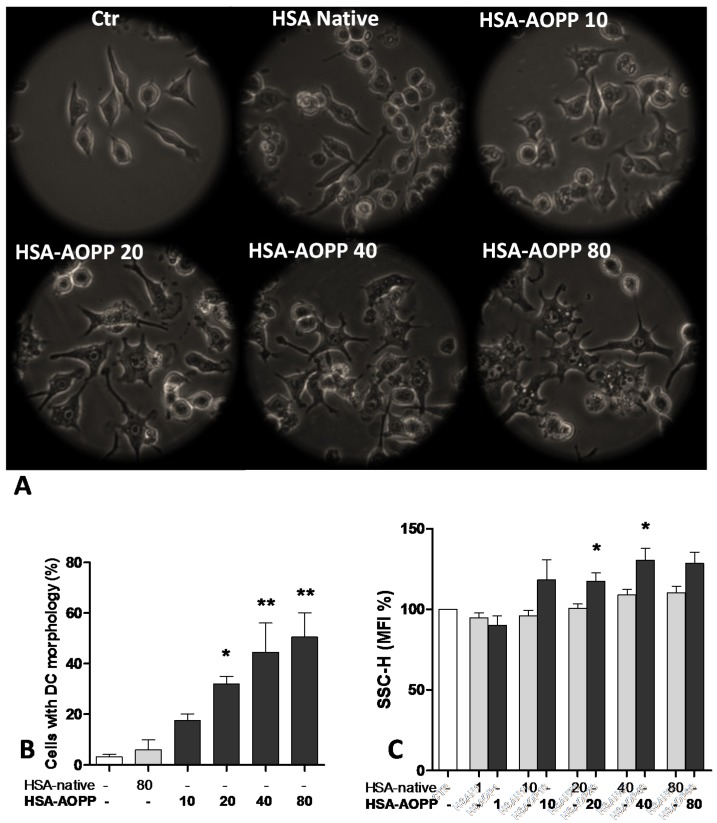
(**A**) Phase contrast microscopy of RAW cells cultured with different HSA-AOPP concentrations (magnification 100×); (**B**) Percentage count of RAW cells observed by phase contrast microscopy. Data represent mean + SE. * *p* < 0.05, ** *p* < 0.01, *** *p* < 0.001 vs. untreated cells; (**C**) Flow cytometric evaluation of RAW cell complexity as a percentage of Mean Fluorescence Intensity (MFI) of side scatter (SSC-H). Data represent mean + SE. * *p* < 0.05 vs. native HSA.

RAW264.7 cells differentiated from a macrophage-like appearance into a dendritic-like aspect following 48 h of incubation with increasing concentrations of HSA-AOPP, as shown by morphological changes assessed by microscopic observation (Figure 1A). Cell count indicated that HSA-AOPP significantly increased the percentage of cells having dendritic morphology (Figure 1B). Native HSA did not induce clear-cut morphological changes. Microscopic evaluation of cells showing dendritic phenotype was consistent with cytometry-assessed morphological parameters. Flow cytometric analysis of the forward scatter (FSC), reflecting the cell size, was not significantly altered by AOPP, while the side scatter (SSC), which indicates cell complexity, increased after AOPP treatment (Figure 1C). At the highest AOPP concentration, the increase in the median SSC cell morphology parameter lacked statistical significance over native HSA. We believe that this may be due to an increase in the SSC induced by the corresponding native HSA concentration against which statistical comparison has been performed, and/or to the appearance of a low but significant degree of apoptosis (highlighted in the hypodiploid DNA data presented in Section 2.4).

### 2.2. CD36 Expression in RAW264.7 Cells and Time Course of Surface DC Markers upon Treatment with HSA-AOPP

**Figure 2:**
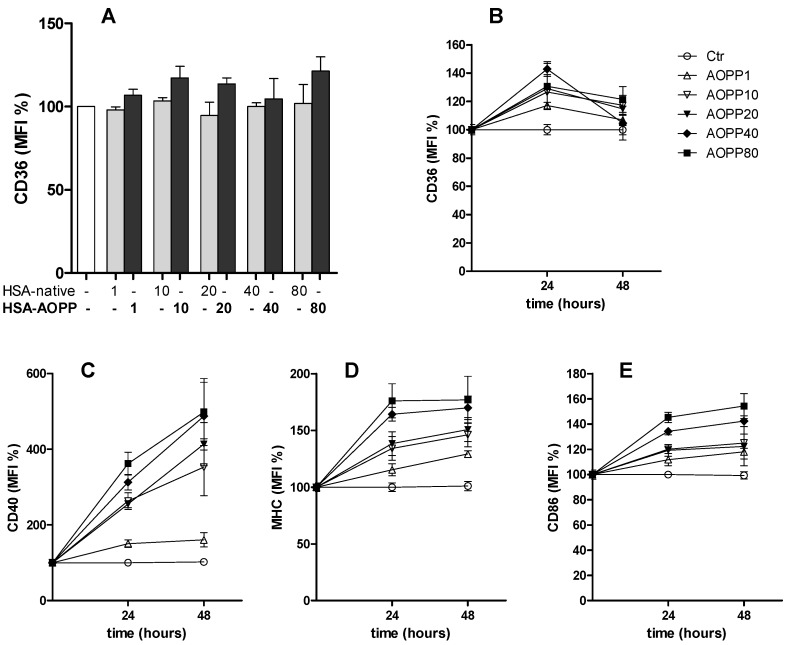
CD36 expression in RAW264.7 cells: (**A**) CD36 analysis of RAW cells treated with HSA-AOPP and with native-HSA; and (**B**–**E**) time course surface expression of CD36, CD40, MHC Class II, and CD86, respectively, in RAW cells treated with HSA-AOPP.

RAW264.7 cells have the features of a macrophage cell line, and show high expression of CD36, a key receptor that is responsible for the uptake of modified low density lipoproteins leading to lipid loading in macrophages and which is an important factor resulting in endoplasmic reticulum (ER) stress [19]. CD36 surface expression did not increase following 48 h of HSA-AOPP treatment (Figure 2A). However, by analyzing the time course of CD36 surface expression following HSA-AOPP treatment, a transient increase was observed at 24 h, that rapidly dropped to near basal levels at the 48-hour interval (Figure 2B). The surface expression of DC markers CD40, MHC Class II and CD86 increased at 24 h and continued to increase up to 48 h (Figure 2C–E). These results suggest that oxidized albumin uptake by CD36 may represent a first step leading to the process of DC differentiation.

### 2.3. HSA-AOPP Induced Phenotypic DC Markers Expression in RAW264.7 Cells. Flow Cytometry of Phenotypic Parameters

**Figure 3:**
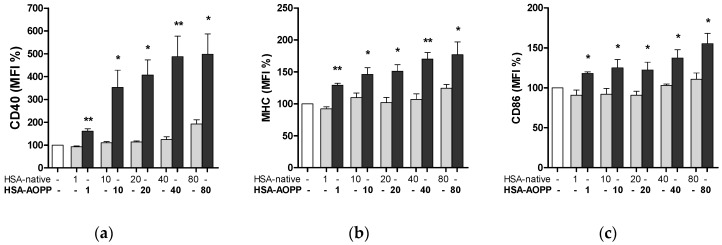
Phenotype analysis, assessed by the DC markers CD40 (**a**); MHC Class II (**b**) and CD86 (**c**), of RAW cells treated with HSA-AOPP and with native-HSA. * *p* < 0.05, ** *p* < 0.01 vs. native-HSA.

Following a 48 h treatment with HSA-AOPP, RAW264.7 macrophages showed an increased expression of markers, thus reflecting commitment to dendritic cell lineage and activation. As shown in Figure 3, HSA-AOPP dose-dependently increased the surface expression of CD40, whose signaling gives rise to upregulation of MHC class II and of co-stimulatory molecule CD86, which are, respectively, markers of DC maturation and activation, thereby rendering them effective antigen-presenting cells [20].

### 2.4. Evaluation of Cell Viability

**Figure 4:**
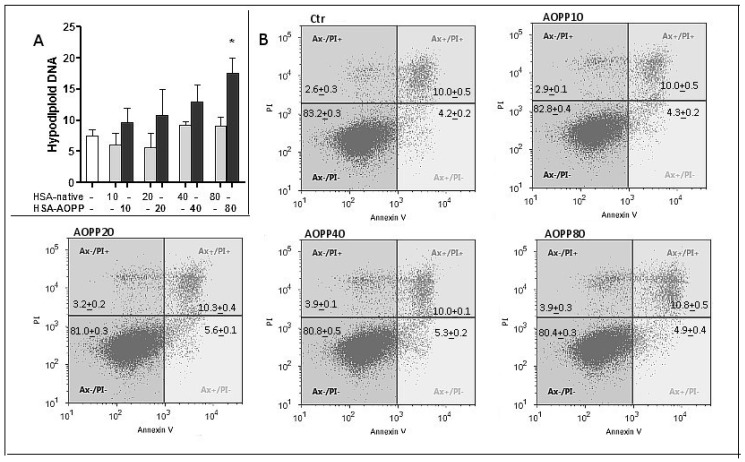
(**A**) Hypodiploid DNA evaluation in RAW264.7 cells treated with HSA-AOPP or native-HSA; and (**B**) flow cytometric Annexin V and Propidium Iodide assay; * *p* < 0.05 vs. native-HSA.

Hypodiploid DNA was evaluated as an index of cell apoptosis. RAW264.7 were treated with a wide range of concentrations of HSA-AOPP though maintaining a sub-toxic level. AOPP-HSA had very little effect on cell viability, even after 48 h of treatment. The apoptotic index as mirrored by hypodiploid DNA evaluation was significantly higher than the levels observed in native-HSA treatment, albeit only at the highest amount that was used (Figure 4A). Even at that concentration, however, the hypodiploid DNA fraction was minimal as compared to living nuclei, suggesting that most cells remained alive and responsive to treatment in terms of both phenotypic and functional DC features. We also evaluated apoptosis using Annexin V and Propidium Iodide (PI) staining. The results reported in Figure 4B do not show any significant increase in either Annexin V positive/PI negative cells or in Annexin V positive/PI positive cells.

### 2.5. Cell Surface Thiol Groups and Intracellular ROS Production Are Modulated by HSA-AOPP

**Figure 5:**
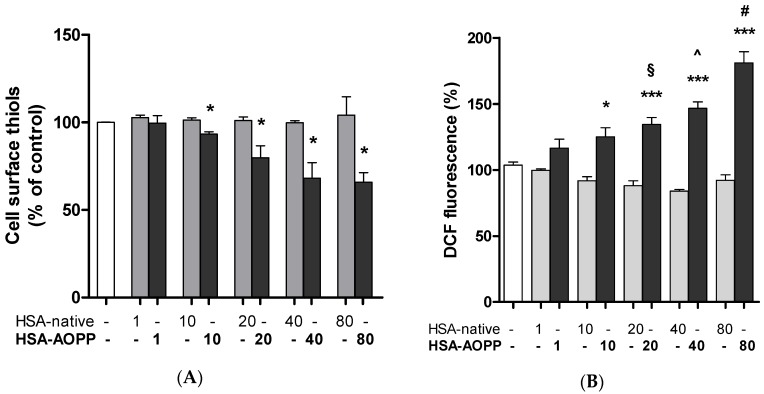
(**A**) Cell surface thiols in cells treated with HSA-AOPP and with native HSA. * *p* < 0.05 vs. HSA-native; (**B**) ROS production in RAW264.7 cells. * *p* < 0.05 vs. untreated control cells and vs. HSA-native 10 nmol/mL; *** *p* < 0.001 vs. untreated control cells; § *p* < 0.01 vs. HSA-native 20 nmol/mL; ^ *p* < 0.001 vs. HSA-native 40 nmol/mL; # *p* < 0.001 vs. HSA-native 80 nmol/mL.

HSA-AOPP treatment of RAW264.7 cells for 2 h induced a dose-dependent decrease of the cell surface thiol pool, as shown by AlexaFluor maleimide fluorescence decrease. On the contrary, changing concentrations of native HSA had no effect on the surface thiol pool (Figure 5A). Similarly, as compared to native HSA, HSA-AOPP induced an increase in intracellular ROS production in RAW264.7 cells evaluated by dichlorofluorescein fluorescence after a 30-min treatment (Figure 5B). Data from Figure 5A,B suggest that there is likely an association between the decrease in cell surface thiols and the increase in intracellular ROS production. An analysis of the correlation resulted in a negative Pearson correlation coefficient of −0.879 (*p* < 0.05).

### 2.6. N-Acetylcysteine (NAC) Pre-Treatment Enhanced the Surface Thiols and Prevented Their AOPP-Dependent Decrease

**Figure 6:**
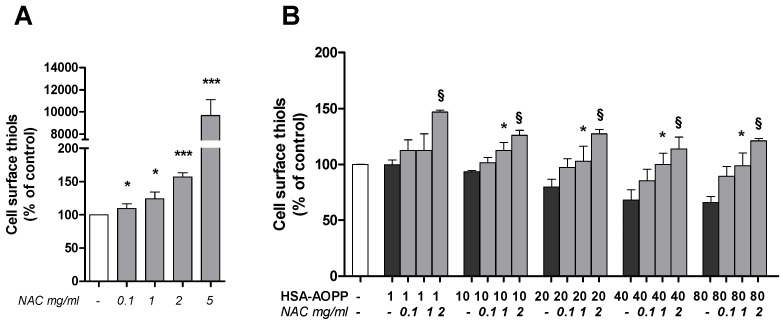
RAW264.7 cell surface thiols upon NAC treatment: (**A**) cells treated with increasing concentrations of NAC; and (**B**) cells pre-treated with NAC and then treated with HSA-AOPP. * *p* < 0.05 and § *p* < 0.01 vs. HSA-AOPP treatment.

Following the observation that AOPP-modified albumin induced thiol loss in the cell surface, we pre-treated RAW264.7 cells with NAC before treating them with HSA-AOPP. NAC treatment (0.1, 1, 2, and 5 mg/mL) dose-dependently increased surface thiols at all concentrations that were used which ranged from 0.1 to 5 mg/mL (Figure 6A). Pre-treatment with NAC before HSA-AOPP exposure starting from the 1 mg/mL concentration prevented the –SH group loss that was induced by HSA-AOPP (Figure 6B). Moreover, AOPP-HSA induced exofacial thiol reduction, even in cells that had been pretreated with NAC and in which membrane thiols were increased, compared to non-pretreated cells.

### 2.7. NAC Pre-Treatment Reduced ROS Production

**Figure 7:**
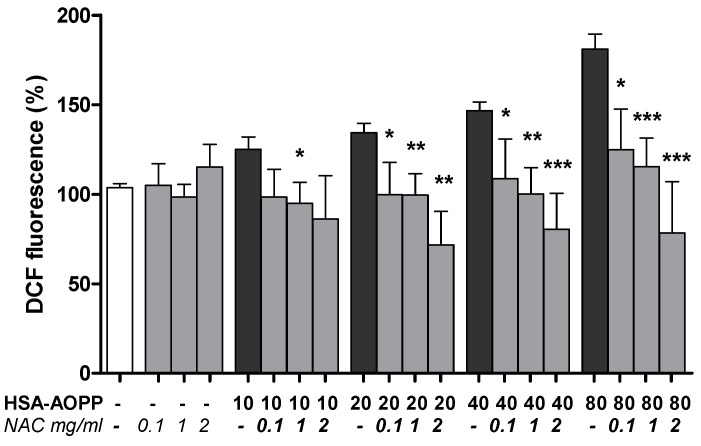
Intracellular ROS production. Cells pre-treated with NAC and then treated with HSA-AOPP. * *p* < 0.05, ** *p* < 0.01 and *** *p* < 0.001 vs. HSA-AOPP treatment.

NAC pre-treatment reduced ROS production induced by HSA-AOPP treatment (Figure 7).

### 2.8. The Effect of NAC Treatment on Cell Surface Markers

**Figure 8:**
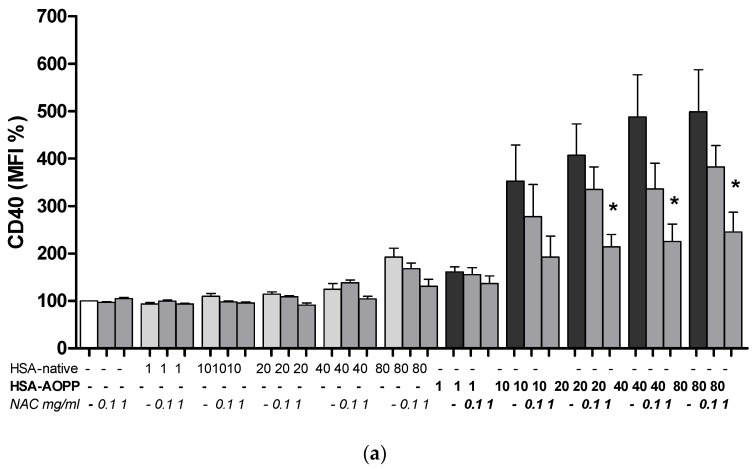

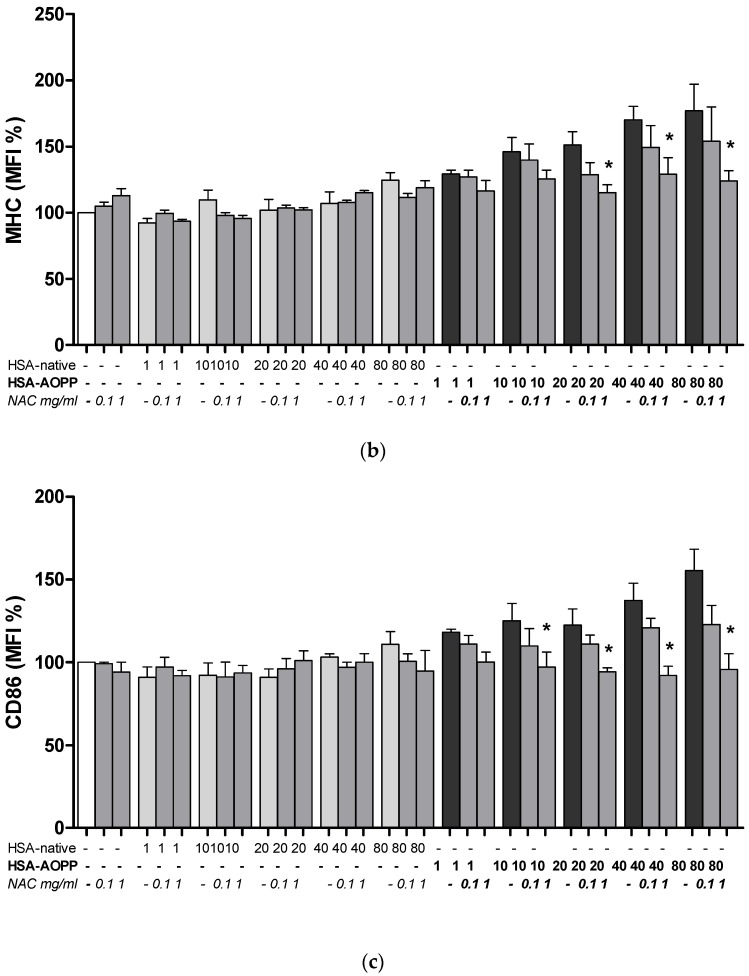
Evaluation of cell surface dendritic markers CD40 (**a**); MHC Class II (**b**) and CD86 (**c**) in RAW264.7 cells following NAC pre-treatment. * *p* < 0.05 vs. HSA-AOPP treatment.

Based on our experimental observations with NAC for cell surface thiol expression and for intracellular ROS production, the concentrations of 0.1 and 1 mg/mL NAC were used to quantify the expression of specific DC surface markers. NAC at the concentration of 1 mg/mL, but not at the lower study concentration, prevented the increase of CD40, CD86 and MHC expression in RAW264.7 cells (Figure 8).

### 2.9. Dendritic Cell Functional Assay. MLR for T-Cell Proliferation Induced by HSA-AOPP Treated RAW264.7 Cells

**Figure 9:**
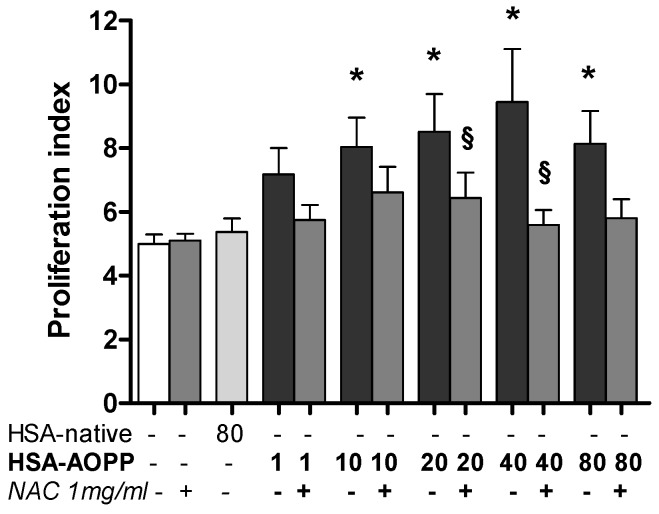
Proliferative T-cell activity stimulation (five days) induced by RAW cells treated or not with AOPP for 48 h.

RAW264.7 cells treated with HSA-AOPP for 48 h and then co-incubated with lymphocytes for five days induced proliferative T-cell activity. NAC pretreatment of RAW264.7 cells abolished T cell proliferation (Figure 9).

### 2.10. p38 MAPKinase and JNK Are Affected by AOPP Treatment of RAW264.7 Cells

**Figure 10:**
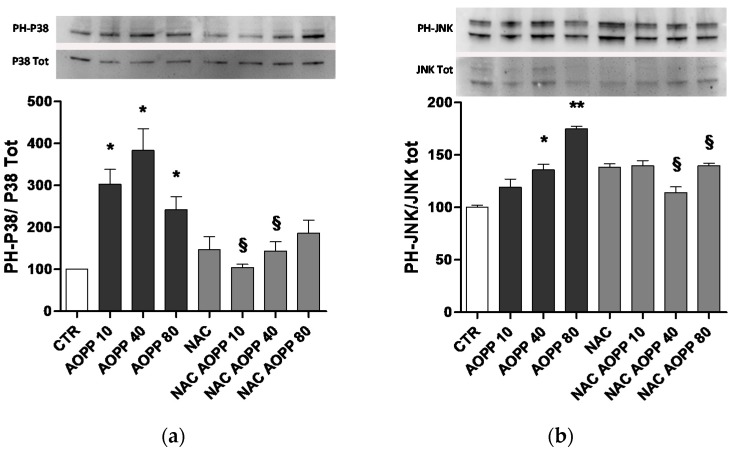
Proliferative p38MAPKinase (**a**) and JNK (**b**) phosphorylation induced in RAW cells by HSA-AOPP. * *p* < 0.05 vs. Ctr; ** *p* < 0.01 vs. Ctr; § *p* < 0.05 vs. corresponding HSA-AOPP treatment.

RAW264.7 cells treated with HSA-AOPP showed an increase in the phosphorylation of p38MAPKinase. Similarly, JNK was phosphorylated by HSA-AOPP, while NAC pretreatment prevented p38MAPKinase and JNK phosphorylation (Figure 10). 

### 2.11. Effects of N-Ethylmaleimide, a Thiol Modifier, on RAW264.7 Cell ROS Production and DC Markers

**Figure 11:**
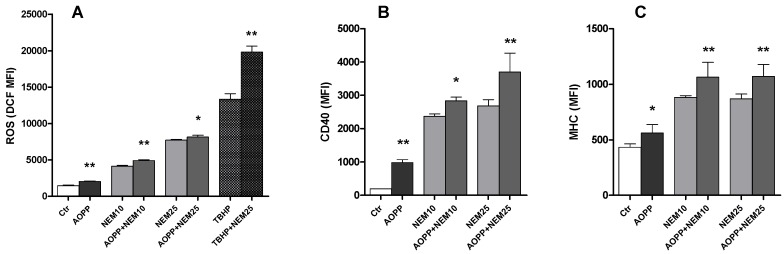
Effect of the thiol modifier *N*-ethylmaleimide on RAW264.7 cells: (**A**) intracellular ROS production; and (**B**,**C**) surface expression of CD40 and MHC class II. * *p* < 0.05 vs. treatment without HSA-AOPP; ** *p* < 0.01 vs. treatment without HSA-AOPP.

To further check the role of thiol groups as a mechanism for DC formation, we treated cells with 10 and 25 µM *N*-ethylmaleimide and we evaluated the effect on intracellular ROS production and on the expression of the DC markers CD40 and MHC class II. *N*-ethylmaleimide strongly increased ROS production when administered alone and strengthened the effect of HSA-AOPP. The free radical inducer Ter-butyl-hydroperoxide (TBHP), that was used as a positive control, was also potentiated by the treatment with *N*-ethylmaleimide (Figure 11A). The surface expression of CD40 and MHC class II was increased by *N*-ethylmaleimide alone; the increase in DC markers induced by AOPP treatment was also potentiated by the thiol modifier (Figure 11B,C).

### 2.12. GSH and GSH/GSSG Ratio Decreased Following Treatment by HSA-AOPP in RAW264.7 Cells

**Figure 12:**
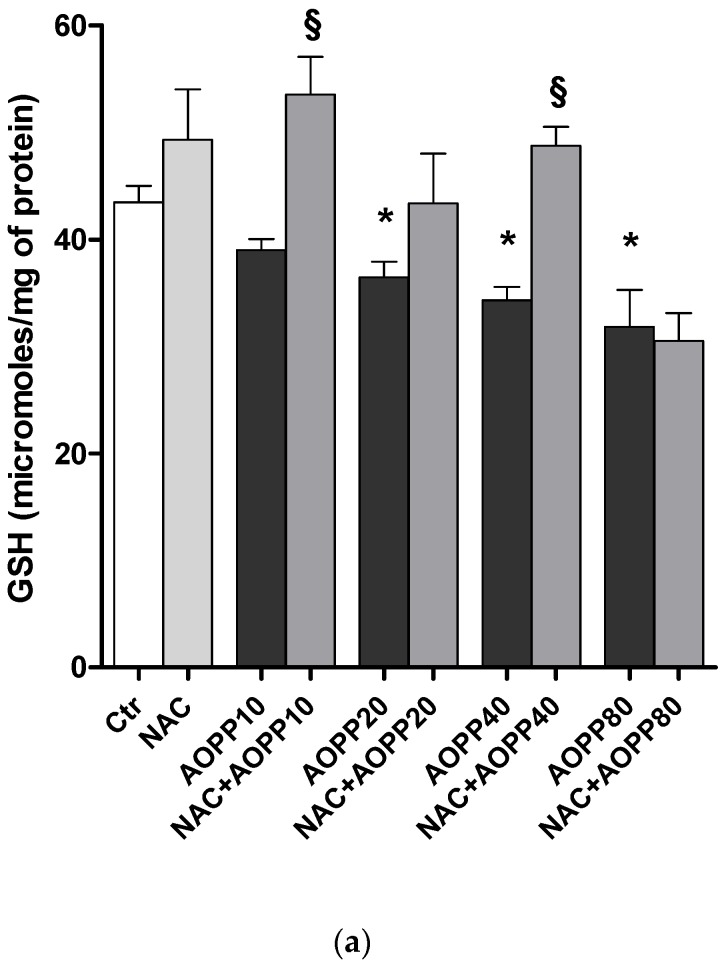

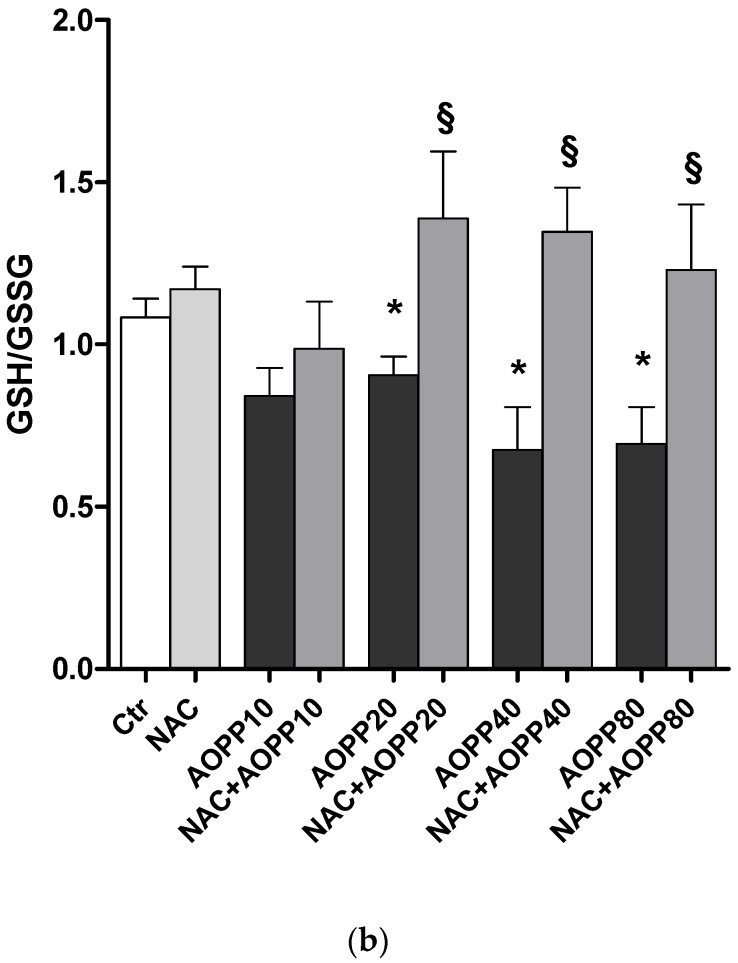
Glutathione (GSH) (**a**) and GSH/GSSG (**b**) evaluation in RAW264.7 cells. *p* < 0.05 vs. Ctr; *p* < 0.05 vs. corresponding AOPP treated cells.

RAW264.7 cells treated with HSA-AOPP for 2 h showed a decrease in glutathione levels and in the ratio between GSH and GSSG. NAC pretreatment prevented GSH and GSH/GSSG decrease (Figure 12).

## 3. Discussion

Our results indicate that AOPP-albumin induces a transition toward DC phenotype in cultured RAW264.7 macrophages. This process involves changes in intracellular ROS production and in cell surface thiol groups. We have also shown that NAC supplementation in cultured RAW264.7 macrophages prevents intracellular ROS production and oxidation of plasma membrane thiols, and blocks HSA-AOPP induced DC differentiation.

The biological relevance of the non-oncotic properties of albumin in blood plasma as a major target for oxidative modifications, in particular for those induced by hypochlorous acid, has already been reported [21,22]. Albumin in plasma has more potent free radical scavenger properties than low molecular weight antioxidants like ascorbic acid [23]. 

Oxidation of albumin induces post-translational modifications that are collectively expressed as AOPP, which not only represent an oxidative stress product and biomarker, but also induce further ROS production in cells as shown elsewhere and confirmed by our results, thus sustaining the existence of a feedback loop [11]. It has been convincingly shown that an excess of oxidative stress is linked to, and promotes, inflammation and immune cell activation. 

AOPP act as uremic toxins on the kidney and other organs and their accumulation is an independent risk factor for cardiovascular events especially but not exclusively in CKD. We previously found that AOPP plasma levels were increased in cardiovascular diseases [24], as well as in pro-atherogenic conditions [25]. 

Given its ability to circulate throughout the body, oxidized albumin acts as a systemic danger signal and/or a damage-associated molecular pattern (DAMP) with immunogenic activity. Along this line, high AOPP plasma levels may contribute to innate immunity dysregulation mediated by an excessive DC differentiation and activation. Herein we provide proof that hypochlorite modified proteins may be immunogenic not only because they are recognized by macrophages and DCs as Biedron et al. previously described, but also because they stimulate and drive the differentiation of DCs from macrophages [26]. 

The complex role played by DCs at both the systemic and local level is dependent on, and finely tuned by, their interaction and overlap with the circulating monocytes and with the macrophages in tissues. As an example, macrophages displaying DC-like features are present in the kidney, but due to the multiplicity of functions and distribution of these cells, at this time we are still far from a clear understanding of their role in modulating or promoting rapid progression of renal damage [27,28].

Recent detection of DCs in atherosclerotic plaque, both in humans and in animal models, is of the utmost interest, given the myriad functions of DCs, which include lipid uptake, antigen presentation, efferocytosis, and inflammation resolution. In particular, CD40, MHC Class II and CD1d DC marker expressions have been found in atherosclerotic lesions [29]. Increased DC differentiation of mononuclear cells collected from patients with acute coronary syndromes point to a role of these cells in specific cardiovascular diseases associated with systemic and local (coronary plaque lesion) inflammatory activity [30]. 

As previously shown by Alderman et al., in vitro AOPP supplementation to DCs differentiated from human monocytes treated with IL-4 and GMCSF increased their capacity to activate T cells, but did not induce the expression of DC maturation markers [31]. 

We found that HSA-AOPP is able to induce a phenotypic transition from macrophages toward cells with typical dendritic morphology. Surface DC markers were increased by AOPP treatment, as proven by the expression of CD40 and of MHC class II which is known as a classical antigen-presenting molecule. In addition, we showed the upregulation of co-stimulatory molecule CD86, which interacts with its receptor CD28 on T cells and is needed for T cell stimulation. Furthermore, these DCs possessed functional DC activity in stimulating the proliferation of T-lymphocytes. The trans-differentiation of macrophages into DCs may represent a source of potent antigen-presenting dendritic cells. We believe that these cells may trigger and drive a series of immune inflammatory reactions that could be potentially detrimental to the vessel wall stability of the atherosclerotic plaque that is present in patients with chronic renal damage. 

Proteins located at the external cell membrane level face an extracellular oxidizing environment and their thiols are mostly oxidized as disulfides. Moreover, the intracellular redox state also regulates the external cell surface thiols, thus intra and extracellular thiols are tightly regulated and their perturbation triggers signaling, leading to cell phenotypic and functional modifications [32,33]. 

There are several biological consequences related to reduced levels of plasma membrane cell thiols. Their loss is associated with the induction of p38 MAP kinase cell signaling, leading to apoptosis in vitro in U937 cells [34]. Moreover, Tanaka et al. found that 1-chloro-2,4-dinitrobenzene and diethyl maleate, which oxidize thiol groups, inhibited the H_2_O_2_-induced phosphorylation of eNOS and Akt [35]. Thiol redox status has been convincingly associated with progression of atherosclerosis [36]. Oxidation of plasma membrane thiols has been proposed as the trigger of an inflammatory mechanism that leads to the onset and progression of atherosclerosis through H_2_O_2_ production, NF-kB activation, enhanced expression of cell-cell adhesion molecules, and attachment of monocytes to endothelial cells [37]. 

We found that the cell surface thiols dose-dependently decreased following HSA-AOPP treatment of RAW264.7 macrophages, suggesting that the –SH group decrease on the cell surface may be part of a signaling mechanism, that together with ROS production, results in the phenotypic modifications of RAW macrophages toward a dendritic phenotype. On the other hand, treatment with NAC recovered surface thiol loss, limited ROS production and hampered the macrophage to dendritic phenotype transition. 

NAC is used in patients with chronic bronchitis, as well as to fight liver damage after acetaminophen poisoning, and to reduce renal damage associated with the use of contrast agent. NAC also plays a role in the modulation of atherosclerotic processes; in experimental models of damage to the arterial wall, NAC was effective in reducing both neointimal thickening promoted by procoagulant activity and oxidative stress generated by balloon injury to normal abdominal aortae and to aortae with neointima [38]. NAC supplementation to nitroglycerin reduced the occurrence of cardiovascular events in 200 patients with unstable angina who were followed-up for four months [39]. The main antioxidant NAC activities are represented by ROS scavenging and by participation in the formation of GSH. 

NAC has many possible mechanisms of action: besides being a free radical scavenger and a substrate for GSH synthesis, it can interfere with thiol redox transition by reducing or limiting the formation of disulfides. It is also likely that NAC treatment on cells acts by directly reducing the cell membrane disulfides. NAC has been shown to keep exofacial surface protein thiols in their reduced form; however, in our opinion, an inside-out activity cannot be excluded in our model, since NAC easily enters cells. 

It is known that in vivo the intracellular milieu is mainly reducing while the extracellular one is mostly oxidizing, and several surface thiols are in the disulfide form. Thus, similarly, in vitro cultured cells seem to have a considerable amount of membrane thiols in the disulfide oxidized form that can be reduced by exogenous thiols from NAC. NAC also enters cells and may influence the intracellular redox status, which in turn participates in the redox status of surface –SH. Therefore, NAC may influence surface thiols both by acting as a regulator of intracellular redox status or as a direct thiol-disulfide reductant at the external cell membrane level. 

We found that NAC treatment dose-dependently increased the cell surface –SH groups. It can be hypothesized that upon HSA-AOPP treatment, both the NAC rescue effects on the loss of plasma membrane thiols and the inhibition of DC-like cell formation are at least partially mediated by NAC thiol-disulfide exchange activity as a reductant. Furthermore, our observation of an initial CD36 increase within 24 h of treatment is consistent with the previous finding by Biedron et al. who suggested that HSA-AOPP acts via its intake through receptors like CD36 or scavenger receptor SRA [26]. It is known that CD36 is a mediator of AOPP damage through pro-oxidant stress dependent mechanisms in different cell types [40]. Thus, we can hypothesize that CD36 may both uptake HSA-AOPP and drive oxidative stress mediated activation of the cell, both of which lead to processing of oxidized albumin and expression of DC markers. Since the receptor mediated mechanism may induce a response as a consequence of HSA-AOPP binding, thereby eliciting downstream signaling, it is possible that NAC also interferes with ROS activity at the intracellular level.

## 4. Conclusions 

Our results highlight that uremic toxins such as plasma AOPP affect innate immune cell phenotype and function by altering the thiol redox equilibrium. Even if DC content at the level of the kidney and in atherosclerotic plaque appears to be negligible, macrophages at these sites are more abundant and may migrate, accumulate and even proliferate in these inflammatory sites. We studied (in vitro) and herein describe a new mechanism related to an excess of oxidized proteins that promotes an imbalance in the thiol-redox equilibrium that might play a role in the rapid plaque progression observed in patients with advanced kidney disease. As a next step, we should verify in humans with kidney disease whether increased amounts of AOPP are associated with specific features of macrophages in atherosclerotic plaque. At this time, we also believe that dissection of thiol redox signaling in innate immune cells modulated by AOPP represents an important research topic which could help us to identify new and more specific therapeutic strategies to be delivered to high-risk CKD patients with coronary and extra-coronary atherosclerosis.

## 5. Materials and Methods 

### 5.1. Materials

Potassium iodide (KI), chloramine-T, human serum albumin, sodium-hypochlorite, *N*-ethylmaleimide and Citric acid monohydrate were purchased from Sigma-Aldrich (Milan, Italy) and phosphate buffer saline (PBS buffer, 0.1 mol/L sodium phosphate, 0.15 mol/L sodium chloride, pH 7.2) were bought from Euroclone.

### 5.2. AOPP Preparation

Human serum albumin (30 mg/mL) was incubated with 100 mM HOCl at room temperature for 30 min. The reaction was stopped by an equimolar concentration of thiosulfate to block excess unreacted HOCl, after which extensive dialysis was carried out for 24 h at 4 °C. 

### 5.3. AOPP Evaluation

AOPP in HSA preparations were evaluated according to the improved method of Hanasand M et al. [41]. Briefly, 40 μL samples were pipetted into a 96-well microplate and 160 μL of 0.20 mol/L citric acid was added. A calibration curve of Chloramine-T standards prepared fresh daily in 0.20 mol/L citric acid and KI (10 μL; 1.16 mol/L in PBS) was added to the standards. The absorbance was read at 340 nm on a Thermo Labsystems Ascent IEMS microplate reader. AOPP concentrations were expressed as nmol/mL of chloramine-T equivalents. 

### 5.4. Cells and Treatments

The RAW264.7 macrophage cell line was from American Type Culture Collection (ATCC, Rockville, MD, USA). In our HSA preparations, AOPP concentrations were 154.04 ± 12.3 nmol/mg of protein in HOCl-treated HSA and 0.81 ± 0.2 nmol/mg in native HSA. In preliminary experiments, to mimic a lower rate of oxidative stress, we oxidized HSA with 20 mM HOCl, which resulted in AOPP concentration of 32.8 ± 5.2 nmol/mg of protein. The effects of the two AOPP preparations (32.8 ± 5.2 and 154.04 ± 12.3 nmol/mg of protein) on the expression of DC surface markers in RAW264.7 cells are shown in Figure S1 in Supplementary material. Cells were treated in complete medium in 6, 12 or 24 wells (depending on the experimental determinations) with increasing AOPP-HSA concentrations ranging from 1 to 80 nmoles/mL of AOPP, corresponding to AOPP levels found in plasma from normal subjects and hemodialysis patients [1]. Native-HSA at concentrations corresponding to those used for AOPP-HSA were also tested. Results are the mean of 3 to 5 experiments.

### 5.5. Flow Cytometry

To assess DC surface markers, cells were stained with antibodies for CD40, CD36, CD86 and MHC (class II) from Life technologies. An Attune acoustic focusing cytometer with attune cytometric software was used to analyze the samples. Exofacial thiols were assessed by AlexaFluor 488-maleimide (Life Technologies,Waltham, MA, USA) staining for 30 min at 37 °C in PBS. For this staining, RAW264.7 cells were treated with NAC for 30 min and then washed 3× with PBS and detached by trypsin. Detached cells were centrifuged and resuspended in PBS containing AlexaFluor 488-maleimide and stained for 30 min. After 2× washings, cells were analyzed on an Attune Acoustic Focusing cytometer. Intracellular ROS production was evaluated by dichlorofluorescein diacetate (DCFH-DA, Sigma-Aldrich, Milan, Italy). Following treatment cells were stained with 5 μM DCFH-DA in PBS for 20 min at 37 °C. Apoptosis was evaluated by flow cytometry using Alexa Fluor 488 Annexin V/Dead Cell Apoptosis Kit (Life Technologies, Waltham, MA, USA).

### 5.6. Western Blotting

Cells were lysated in RIPA buffer and run on reducing SDS-acrylamide gels. Samples were electrotransferred to PVDF, and the membranes were saturated at room temperature for 1 h, incubated with primary antibodies overnight, and then with the corresponding horseradish peroxidase-secondary antibodies (Santa Cruz, Heidelberg, Germany) for 1 h at room temperature. The bands were visualized by ECL chemiluminescence (Pierce,Monza, Italy). Bands were quantified by optical densitometry.

### 5.7. Glutathione Evaluation

Glutathione (GSH and GSSG) was evaluated as described elsewhere [42]. After treatment, cells were detached and harvested in a PBS solution and about 25% of the cell suspension was used for protein dosage, the remaining 75% of the cell suspension was mixed with the same volume of the precipitating solution (2 mM EDTA, 0.61 N TCA and 0.02 N HCl) and centrifuged (Euroclone,Milan, Italy) at 4000 rpm for 15 min. at 4 °C. The resulting supernatant was used to evaluate the GSH and GSSG contents by the reaction with o-phthalaldehyde (OPA). Standard solutions were prepared by dissolving GSH or GSSG in Redox Quenching Buffer (RQB) containing 20 mM HCl, 5 mM DTPA and 10 mM ascorbic acid. Samples or standards (GSH or GSSG) were treated with 5% TCA-RQB solution. To evaluate GSSG levels, *N*-ethylmaleimide (7.5 mM in RQB) was added. Subsequently, 1 M potassium phosphate (KPi) buffer (pH 7.0) was added to all tubes and the samples were incubated for 5 min. at room temperature. For the GSSG assay, 100 mM dithionite-RQB was used and the samples were incubated for 60 min. at room temperature following which 0.1 M KPi buffer (pH 6.9) and OPA (5 mg/mL in methanol) were added. After a 30-min incubation at room temperature, sample fluorescence was monitored by a Perkin Elmer fluorimeter (Perkin Elmer Life and Analytical Sciences, Shelton, CT, USA) at 365/430 nm.

### 5.8. Mixed Leukocyte Reactions (MLRs)

For the MLRs utilizing mouse cells, 2 × 10^5^ CFSE (2 μM)-labeled allogeneic (BL6) lymphocytes CD4+ cells (responders) were cultured with RAW264.7 cells (stimulators) treated or not with HSA-AOPP. Cells were plated in microplates (Corning,NY, USA) at S:R ratios of 1:5, 1:10 and 1:100. Proliferation of alive (propidium-iodide negative) CD3+CD4+ cells was evaluated by flow cytometry as CFSE dilution after a 5-day incubation.

### 5.9. Statistical Analysis

Data were expressed as mean ± SE. Differences between means were compared by Student’s *t* test. A *p* < 0.05 was considered statistically significant. 

## Supplementary Materials

The following are available online at www.mdpi.com/2072-6651/9/1/27/s1, Figure S1: Effects of AOPP concentration of 32.8 ± 5.2 and 154.04 ± 12.3 (nmol/mg of protein) on the expression of DC surface markers. * *p* < 0.05 vs. Ctr; ** *p* < 0.01 vs. Ctr.

## References

[1] Witko-Sarsat V., Friedlander M., Capeillere-Blandin C., Nguyen-Khoa T., Nguyen A.T., Zingraff J., Jungers P., Descamps-Latscha B. (1996). Advanced oxidation protein products as a novel marker of oxidative stress in uremia. Kidney Int..

[2] Capeillere-Blandin C., Gausson V., Descamps-Latscha B., Witko-Sarsat V. (2004). Biochemical and spectrophotometric significance of advanced oxidized protein products. Biochim. Biophys. Acta.

[3] Witko-Sarsat V., Descamps-Latscha B. (1997). Advanced oxidation protein products: Novel uraemic toxins and pro-inflammatory mediators in chronic renal failure?. Nephrol. Dial. Transplant..

[4] Witko-Sarsat V., Gausson V., Descamps-Latscha B. (2003). Are advanced oxidation protein products potential uremic toxins?. Kidney Int..

[5] Kaneda H., Taguchi J., Ogasawara K., Aizawa T., Ohno M. (2002). Increased level of advanced oxidation protein products in patients with coronary artery disease. Atherosclerosis.

[6] Cakatay U. (2005). Protein oxidation parameters in type 2 diabetic patients with good and poor glycaemic control. Diabetes Metab..

[7] Shi X.Y., Hou F.F., Niu H.X., Wang G.B., Xie D., Guo Z.J., Zhou Z.M., Yang F., Tian J.W., Zhang X. (2008). Advanced oxidation protein products promote inflammation in diabetic kidney through activation of renal nicotinamide adenine dinucleotide phosphate oxidase. Endocrinology.

[8] Servettaz A., Guilpain P., Goulvestre C., Chéreau C., Hercend C., Nicco C., Guillevin L., Weill B., Mouthon L., Batteux F. (2007). Radical oxygen species production induced by advanced oxidation protein products predicts clinical evolution and response to treatment in systemic sclerosis. Ann. Rheum. Dis..

[9] Descamps-Latscha B., Witko-Sarsat V., Nguyen-Khoa T., Nguyen A.T., Gausson V., Mothu N., London G.M., Jungers P. (2005). Advanced oxidation protein products as risk factors for atherosclerotic cardiovascular events in nondiabetic predialysis patients. Am. J. Kidney Dis..

[10] Ito S., Yoshida M. (2014). Protein-bound uremic toxins: New culprits of cardiovascular events in chronic kidney disease patients. Toxins.

[11] Witko-Sarsat V., Gausson V., Nguyen A.T., Touam M., Drüeke T., Santangelo F., Descamps-Latscha B. (2003). AOPP-induced activation of human neutrophil and monocyte oxidative metabolism: A potential target for N-acetylcysteine treatment in dialysis patients. Kidney Int..

[12] Witko-Sarsat V., Friedlander M., Capeillère-Blandin C., Nguyen-Khoa T., Nguyen A.T., Zingraff J., Jungers P., Descamps-Latscha B. (1998). Advanced oxidation protein products as novel mediators of inflammation and monocyte activation in chronic renal failure. J. Immunol..

[13] Merad M., Sathe P., Helft J., Miller J., Mortha A. (2013). The dendritic cell lineage: Ontogeny and function of dendritic cells and their subsets in the steady state and the inflamed setting. Annu. Rev. Immunol..

[14] Hespel C., Moser M. (2012). Role of inflammatory dendritic cells in innate and adaptive immunity. Eur. J. Immunol..

[15] Dopheide J.F., Zeller G.C., Kuhlmann M., Girndt M., Sester M., Sester U. (2015). Differentiation of Monocyte Derived Dendritic Cells in End Stage Renal Disease is Skewed towards Accelerated Maturation. Adv. Clin. Exp. Med..

[16] Heymann F., Meyer-Schwesinger C., Hamilton-Williams E.E., Hammerich L., Panzer U., Kaden S., Quaggin S.E., Floege J., Gröne H.J., Kurts C. (2009). Kidney dendritic cell activation is required for progression of renal disease in a mouse model of glomerular injury. J. Clin. Investig..

[17] Kitching A.R. (2014). Dendritic cells in progressive renal disease: Some answers, many questions. Nephrol. Dial. Transplant..

[18] Turell L., Radi R., Alvarez B. (2013). The thiol pool in human plasma: The central contribution of albumin to redox processes. Free Radic. Biol. Med..

[19] Mellman I., Steinman R.M. (2001). Dendritic cells: Specialized and regulated antigen processing machines. Cell.

[20] Seimon T.A., Nadolski M.J., Liao X., Magallon J., Nguyen M., Feric N.T., Koschinsky M.L., Harkewicz R., Witztum J.L., Tsimikas S. (2010). Atherogenic lipids and lipoproteins trigger CD36-TLR2-dependent apoptosis in macrophages undergoing endoplasmic reticulum stress. Cell Metab..

[21] Himmelfarb J., McMonagle E. (2001). Albumin is the major plasma protein target of oxidant stress in uremia. Kidney Int..

[22] Anraku M., Chuang V.T., Maruyama T., Otagiri M. (2013). Redox properties of serum albumin. Biochim. Biophys. Acta.

[23] Pattison D.I., Hawkins C.L., Davies M.J. (2009). What Are the Plasma Targets of the Oxidant Hypochlorous Acid? A Kinetic Modeling Approach. Chem. Res. Toxicol..

[24] Barsotti A., Fabbi P., Fedele M., Garibaldi S., Balbi M., Bezante G.P., Risso D., Indiveri F., Ghigliotti G., Brunelli C. (2011). Role of advanced oxidation protein products and Thiol ratio in patients with acute coronary syndromes. Clin. Biochem..

[25] Spallarossa P., Garibaldi S., Barisione C., Ghigliotti G., Altieri P., Tracchi I., Fabbi P., Barsotti A., Brunelli C. (2008). Postprandial serum induces apoptosis in endothelial cells: Role of polymorphonuclear-derived myeloperoxidase and metalloproteinase-9 activity. Atherosclerosis.

[26] Biedroń R., Konopiński M.K., Marcinkiewicz J., Józefowski S. (2015). Oxidation by neutrophils-derived HOCl increases immunogenicity of proteins by converting them into ligands of several endocytic receptors involved in antigen uptake by dendritic cells and macrophages. PLoS ONE.

[27] Nelson P.J., Rees A.J., Griffin M.D., Hughes J., Kurts C., Duffield J. (2012). The renal mononuclear phagocytic system. J. Am. Soc. Nephrol..

[28] Teteris S.A., Engel D.R., Kurts C. (2011). Homeostatic and pathogenic role of renal dendritic cells. Kidney Int..

[29] Ozmen J., Bobryshev Y.V., Lord R.S. (2001). CD40 co-stimulatory molecule expression by dendritic cells in primary atherosclerotic lesions in carotid arteries and in stenotic saphenous vein coronary artery grafts. Cardiovasc. Surg..

[30] Sharma R., Li D., Zeng Q., Feng Y., Li Y., Wang X., Chao L.S., Tian Y. (2004). Differentiation of dendritic cells in monocyte cultures isolated from patients with unstable angina. Int. J. Cardiol..

[31] Alderman C.J., Shah S., Foreman J.C., Chain B.M., Katz D.R. (2002). The role of advanced oxidation protein products in regulation of dendritic cell function. Free Radic. Biol. Med..

[32] Sahaf B., Heydari K., Herzenberg L.A., Herzenberg L.A. (2003). Lymphocyte surface thiol levels. Proc. Natl. Acad. Sci. USA.

[33] Hirota M., Motoyama A., Suzuki M., Yanagi M., Kitagaki M., Kouzuki H., Hagino S., Itagaki H., Sasa H., Kagatani S. (2010). Changes of cell-surface thiols and intracellular signaling in human monocytic cell line THP-1 treated with diphenylcyclopropenone. J. Toxicol. Sci..

[34] Filomeni G., Rotilio G., Ciriolo M.R. (2003). Glutathione disulfide induces apoptosis in U937 cells by a redox-mediated p38 MAP kinase pathway. FASEB J..

[35] Tanaka T., Nakamura H., Yodoi J., Bloom E.T. (2005). Redox regulation of the signaling pathways leading to eNOS phosphorylation. Free. Radic. Biol. Med..

[36] Ashfaq S., Abramson J.L., Jones D.P., Rhodes S.D., Weintraub W.S., Hooper W.C., Vaccarino V., Harrison D.G., Quyyumi A.A. (2006). The relationship between plasma levels of oxidized and reduced thiols and early atherosclerosis in healthy adults. J. Am. Coll. Cardiol..

[37] Go Y.M., Jones D.P. (2005). Intracellular proatherogenic events and cell adhesion modulated by extracellular thiol/disulfide redox state. Circulation.

[38] Ghigliotti G., Mereto E., Eisenberg P.R., Martelli A., Orsi P., Sini D., Spallarossa P., Olivotti L., Brunelli C. (2001). *N*-acetyl-cysteine reduces neointimal thickening and procoagulant activity after balloon-induced injury in abdominal aortae of New Zealand white rabbits. Thromb. Haemost..

[39] Demicheli G., Zanini P., Bertocchi F., Falcone C., Ghio S., Marinoni G., Montemartini C., Mussini A. (1997). Effect of transdermal nitroglycerin or *N*-acetylcysteine, or both, in the long-term treatment of unstable angina pectoris. J. Am. Coll. Cardiol..

[40] Cao W., Xu J., Zhou Z.M., Wang G.B., Hou F.F., Nie J. (2013). Advanced oxidation protein products activate intrarenal renin-angiotensin system via a CD36-mediated, redox-dependent pathway. Antioxid. Redox Signal.

[41] Hanasand M., Omdal R., Norheim K.B., Gøransson L.G., Brede C., Jonsson G. (2012). Improved detection of advanced oxidation protein products in plasma. Clin. Chim. Acta.

[42] Colla R., Izzotti A., De Ciucis C., Fenoglio D., Ravera S., Speciale A., Ricciarelli R., Furfaro A.L., Pulliero A., Passalacqua M. (2016). Glutathione-mediated antioxidant response and aerobic metabolism: Two crucial factors involved in determining the multi-drug resistance of high-risk neuroblastoma. Oncotarget.

