# Supplementary material for: Advanced Oxidation Protein Products-Modified Albumin Induces Differentiation of RAW264.7 Macrophages into Dendritic-Like Cells Which Is Modulated by Cell Surface Thiols
Source: Toxins (Basel). 2017 Jan 10;9(1):27. doi: 10.3390/toxins9010027 (PMC5308259; doi:10.3390/toxins9010027)
Supplement: Supplementary file 1 [file toxins-09-00027-s001.pdf]

Supplementary Materials: Advanced Oxidation Protein Products-Modified Albumin Induces Differentiation of RAW264.7 Macrophages into Dendritic-Like Cells Which Is Modulated by Cell Surface Thiols

Silvano Garibaldi, Chiara Barisione, Barbara Marengo, Pietro Ameri, Claudio Brunelli, Manrico Balbi and Giorgio Ghigliotti

In setting experiments, we evaluated also other concentrations of AOPP; we used HSA treated with a relatively lower concentration of HOCl (20 mM).

We found that 20 mM and 100 mM HOCl treated HSA resulted in the AOPP concentration (nmol/mg of protein) of 32.8 + 5.2 and 154.04 + 12.3, respectively that reflect conditions of moderate and of high rate of oxidation as found in uremic patients. When treating RAW cells, both these AOPP preparations showed qualitatively similar effects, with quantitative differences (Figure S1).

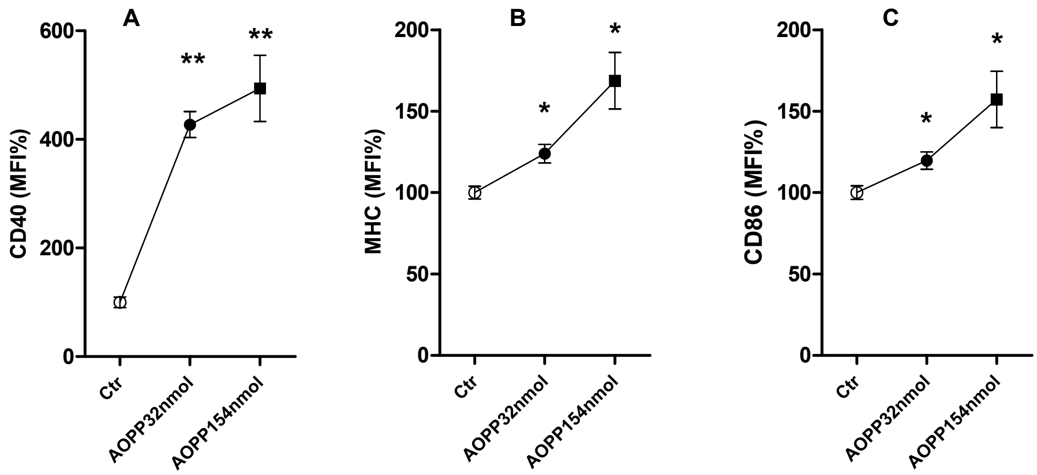

Figure S1. Effects of AOPP concentration of 32.8 ± 5.2 and 154.04 ± 12.3 (nmol/mg of protein) on the expression of DC surface markers CD40 (**A**), MHC ClassII (**B**) and CD86 (**C**). * $p < 0.05$ vs. Ctr; ** $p < 0.01$ vs. Ctr.
